# Does prevention for Alzheimer’s disease exist?

**DOI:** 10.1590/S1980-57642009DN30300006

**Published:** 2009

**Authors:** Sonia Maria Dozzi Brucki

**Affiliations:** MD, PhD, Neurologist from the Cognitive and Behavioral Neurology Group (University of São Paulo); Psychobiology Department (Federal University of São Paulo); Hospital Santa Marcelina, São Paulo, SP, Brazil.

**Keywords:** Alzheimer’s disease, prevention, physical exercise, statins, cholinesterase inhibitors, nutrition

## Abstract

The prevention of Alzheimer’s disease is a growing public health concern amidst an ageing population. Meanwhile, there is no effective or curative treatment available where prevention could greatly reduce health costs. This review was based on reports of potential preventive factors, including modifiable lifestyle factors, as well as preventive pharmacological strategies. Although the present review was not systematic, the reports selected from PubMed using “Alzheimer’s disease” and “prevention” as key-words, allow us to affirm that pursuing a healthy lifestyle; physical, cognitive, leisure activities; good social engagement; a high consumption of fish, low consumption of dietary fat and moderate consumption of wine, and control of vascular risk factors appear to be potential factors for delaying dementia.

## Abstract (pt)

A prevenção da doença de Alzheimer é um problema de saúde pública, enquanto a população está envelhecendo. Por enquanto, não há tratamento efetivo ou curativo e prevenção poderia evitar grandes custos financeiros. Esta revisão foi baseada em estudos sobre potenciais fatores preventivos, incluindo fatores modificáveis de estilo de vida, bem como estratégias farmacológicas preventivas. Embora esta revisão não seja sistemática, estudos selecionados do PubMed, com palavras-chave “Alzheimer’s disease” e “ prevention”, permitem afirmar que ter um estilo de vida saudável, atividades físicas, cognitivas e de lazer; bom engajamento social; aumento no consumo de peixes, redução no consumo de gorduras consumo meoderado de vinho; controle dos fatores de risco vasculares parecem ter vantagens potenciais em retardar a demência.

Delaying the onset of dementia syndrome is a serious health issue which has financial, economic, and social repercussions. The global prevalence of dementia was estimated at 24.3 million in 2001,^1^ while that of Alzheimer’s disease (AD) was 26.55 million in 2006, and is predicted to rise to 106.2 millions by 2050. Retarding the clinical picture of dementia by 12 months represents a huge reduction of around 9.2 million cases.

In Brazil and Latin America, the prevalence of dementia in subjects aged 65 years or older is 7.2% and 15.7% among non-illiterates and illiterates, respectively.^2^

Some risk factors for developing AD are well established: age, family history, female gender (not for all surveys), low educational level, depressive symptoms, traumatic brain injury, the presence of E4 allele of APOE, hypercholesterolemia, smoking, sedentary life-style, and mild cognitive impairment.

Studies have established some factors associated to maintenance of good cognitive aging. In a longitudinal follow-up of a cohort of elderly females over a 15-year period, Barnes et al. observed that persons who remained cognitively stable presented a lesser number of clinical co morbidities (such as arterial hypertension and diabetes), less complaints regarding difficulties in activities of daily living, and greater social integration.^3^ More recently, in an eight year follow-up study of 3075 elders aged between 70 and 79 years in 1997, 30% of the sample had no cognitive decline, while multivariate analysis found the following baseline variables to be associated with being a maintainer: lower age, white race, higher educational and literacy levels, weekly moderate to vigorous exercise and non-smoker status.^4^

Autopsy series of elderly subjects with normal cognitive function have found high burdens of pathological lesions associated with AD, for which several explanations have been proposed. One explanation points to compensatory mechanisms of brain reserve. In the study by Erten-Lyons et al., some factors associated with resistance to dementia despite high AD pathology were determined by comparing individuals with high burden pathology and normal cognition against patients with AD presenting an equivalent burden of lesions. In multiple regression analysis, hippocampal and total brain volumes were significantly larger in the group with normal cognitive functions. The authors proposed three possible explanations: larger brain volumes may indicate a greater preexisting brain reserve; the cognitively intact group may have had other forms of compensation or protection from the pathologic processes; and, the brain volume loss in the AD group may not have been directly caused by neurofibrillary tangles and senile plaques. The underlying protective process remains unknown.^5^

The aim of this report was to describe some results of interventions intended to prevent conversion from healthy or mild cognitive impairment subjects into AD. Some interventions can modify outcome in terms of developing dementia for a certain period of time. Epidemiologic research has revealed various possible preventive factors, including modifiable lifestyle factors and pharmacological strategies. Our aim was to present the results of several clinical trials demonstrating efficacy in delaying progression to dementia. Only results from recent longitudinal studies, randomized controlled trials, and reviews assessing the prevention of AD, conversion of MCI to dementia will be described, where only PubMed articles with full-text in the English language published from 1998 to 2009 (June) using Alzheimer’s Disease and prevention as key-words were selected.

## Non-pharmacological interventions

### Cognitive, leisure, and physical activities

Some studies have found a positive association between leisure activities and lower risk for developing AD or dementia in general.^6,7^ Verghese et al. have attempted to control for possible confounding factors such as the preclinical stage of dementia (which prevents elderly from performing leisure and cognitive activities). They evaluated community-dwelling subjects aged 75 to 85 years old during a period spanning from 1980 to 2001. Subjects were analyzed regarding participation in cognitive activities (reading books or newspapers, writing for pleasure, doing cross-word puzzles, playing board games or cards, participating in group discussions, and playing musical instruments) and for physical activities (playing tennis or golf, swimming, bicycling, dancing, participating in group exercises, playing team games, walking for exercise, climbing more than two flights of stairs, doing housework, or babysitting). At the end of the follow-up (median time of 5.1 years), 124 subjects had developed dementia (AD, n=61; vascular dementia, n=30). Subjects who became demented tended to be older, had less schooling, and performed less cognitive activities (which reduce risk of AD, vascular dementia and mixed dementia), but not physical activities (with the exception of dancing). Among cognitive activities, reading, playing board games, and playing musical instruments were associated with a lower risk of dementia.^8^ In a new analysis of this same sample performed to verify the influence of leisure or physical activity participation on risk of developing amnestic mild cognitive impairment (aMCI), the authors observed that cognitive activities were associated with lower risk of aMCI.^9^ In a study of twin pairs of male veterans (*Duke Twin Study of Memory in Aging*), cognitive activity reduced development of dementia in 26% (particularly among homozygotic pairs – 30% reduction). Physical activity however, played no modifying role.^10^ Social activity is associated with risk for AD, with subjects that have low social engagement being more prone to developing dementia, while those exhibiting mid and late-life social activity were associated with greater cognitive and physical health.^11-14^ Cognitive activities are associated with greater and preserved cognitive reserve, that is, the capacity of the brain to cope with the pathology of dementia through structural or functional characteristics and networks, or alternative cognitive strategies that protect the subject until a certain threshold is reached.

More recently, in a 24-week randomized controlled clinical trial, physical activity was associated with a lower risk for developing MCI and dementia among adults with subjective memory impairment. The study was conducted at the Royal Perth Hospital (Australia) in volunteers aged 50 years or older, randomly assigned to an education program or to a 24-week home-based program of physical activity. There was a significant, albeit modest difference in cognitive performance among those undertaking the physical program, a difference which persisted over an 18-month follow-up period.^15^ The literature on associations among physical activity, cognition, and risk for dementia is conflicting, but this latter study appears to demonstrate a clear positive effect.

In animal studies, physical activity stimulates angiogenesis, synaptogenesis, and neurogenesis. Rats performing treadmill running activity had more astrocytes and neuroblasts with proliferative ability in the subgranular zone of the dentate gyrus of the hippocampus, as well as an increased number of neurons in the transient stage that control rats.^16^ Physical exercise also decreases risk factors for vascular diseases, and can release hormonal factors that boost neuronal functions, reinforcing the theoretical basis of potential benefits.

### Alcoholic beverages

There is a large body of evidence suggesting that moderate alcohol consumption is associated with reduced risk of coronary heart disease, ischemic stroke and dementia.^17-20^ Nineteen longitudinal cohort studies compared relative risk factors for either cognitive decline or dementia in older moderate alcohol consumers versus abstainers/never drinkers. In these studies, approximately 54% of the risk factors were significantly lower in moderate consumers. In a Swedish study, women were followed for 34 years, and analysis was made verifying different types of beverages (wine, beer, and spirits). Wine was protective for dementia, and the association was strongest among women who consumed wine only, and among smokers. Notwithstanding, consumption of spirits at baseline was associated with slightly increased risk of dementia.^21^

Protective mechanisms involved in the association of alcohol consumption with decrease in risk of dementia and vascular alterations, include increase of HDL and decrease of LDL, resulting in lowering of risk due to cholesterol; reduction of insulin resistance and increase in insulin sensitivity; reduction of blood homocysteine; decrease of systemic arterial pressure; reduction in platelet aggregation and fibrinogen levels, with increased fibrinolysis; and reduction in inflammatory markers. Reduction of dementia is associated not only with lower vascular factors, but also with antioxidant polyphenols such as resveratrol. There appears to be an antagonist effect on NMDA receptors by alcohol, and anti-amyloidogenic activity by resveratrol. It has no effect on β- e γ- secretases, but instead promotes intracellular degradation of Aβ via a mechanism that involves the proteasome^20,22,23^. Although mild to moderate consumption (one to two drinks, <30 g/d) is associated with lower risk for cerebrovascular and cardiac diseases; intake of three or more drinks increases rate of ischemic and hemorrhagic stroke.

### Mediterranean diet

The Mediterranean diet is characterized by high intake of fish, vegetables, legumes, fruit, cereal, and unsaturated fatty acids (olive oil); low intake of dairy products, meat, and saturated fatty acids, and moderate consumption of alcohol. Some studies have verified a protective factor of high Mediterranean diet adherence against MCI development (borderline reduction risk) and a reduced risk of MCI conversion to AD.^24-26^

## Pharmacological interventions

### Ginkgo Biloba

Recently, a randomized double blind placebo controlled trial (*G biloba* 240 mg/d *versus* placebo) was published involving five centers in the USA, conducted between 2000 and 2008, with a median follow-up of 6.1 years. Subjects were evaluated every 6 months and 523 individuals developed dementia (246 from placebo group versus 277 from *G biloba* group). Results have shown that Ginkgo Biloba provided no benefit in reducing AD development or conversion of MCI into AD^27^.

### Statins

Four large cohort studies showed the same effect of reduced risk of incident AD with statin use.^28-31^ Some doubts existed over which type of statin was most linked to decreased incidence of dementia based on their lipophilicity. Lipophilic statins were thought to pass the blood-brain barrier (BBB) more efficiently than hydrophilic statins. Haag et al. evaluated the use of cholesterol-lowering drugs and divided them into three classes: lipophilic statins (simvastatin, atorvastatin, cerivastatin), hydrophilic statins (pravastatin, fluvastatin, rosuvastatin), and non-statin cholesterol lowering drugs (fibrates, bile acid binding resins or nicotinic acid and derivatives). Use of statins, despite their lipophilicity, but not use of non-statin cholesterol lowering drugs, was associated with a reduced risk of AD compared with never users.^31^ This protection was effective independent of APOE4 genotype status. These results seem to indicate that permeability of the BBB is not the main protection mechanism. Other mechanisms must be associated to reduced risk of dementia by statins: inhibition of cholesterol synthesis, endothelial functioning, atherosclerosis and oxidative stress reactions, inhibition of amyloid synthesis and reduction of neurofibrillary tangle burden.^31^

### Antihypertensive drugs

There is relatively strong evidence that hypertension in midlife represents a risk factor for dementia or cognitive decline. Recently, a neuropathologic study reported that persons who were treated for hypertension in midlife were less demented clinically and had less AD pathology than both hypertensive patients who were not treated and non-hypertensive patients.^32^ The association of dementia with late-life blood pressure has yielded heterogeneous results. The effects of antihypertensive treatment in late life on risks of dementia are not uniform. It is known however, that arterial hypotension in late life can result in increased risk for cognitive impairment, through ischemia and hypoperfusion. A recent study has shown antihypertensive use to be associated with a significant reduction in dementia in subjects less than 75 years old.^33^

Some randomized controlled trials of antihypertensive medications have shown reductions in incident dementia.^34-36^ Doubts over response to specific classes of antihypertensive drugs remain.

### Cholinesterase inhibitors

The use of cholinesterase inhibitors (donepezil, rivastigmine, galantamine) is well established for AD treatment, with relative increase or preservation of cognitive scores and improvements in measures of global clinical impression observed in studies involving patients with mild to moderate and moderate to severe stages of dementia. Mild cognitive impairment (MCI) is a recognized risk factor for AD development, particularly in amnestic presentation. Some studies, majority of which produced negative results, have been conducted to determine reduction in conversion of MCI to AD.

### Donepezil

During a mean follow-up of three years, results showed no decrease in risk for conversion of MCI to AD upon use of donepezil compared to placebo, although a reduced risk for APOE4 carriers was identified.^37^ In a later sub-analysis of the same sample, a lessened benefit in conversion to dementia was observed among individuals with concomitant depression.^38^

In a randomized double blind placebo controlled clinical trial, with a follow-up of 48 weeks, a discrete improvement in scores on the modified ADAS-Cog (primary measure of efficacy) was found among MCI patients versus the placebo group. Nevertheless, no differences were observed in measures of global or functional impairments. A higher number of subjects in the active group abandoned treatment due to adverse events;^38^ a similar result was demonstrated by Salloway et al.^39^

### Rivastigmine

The InDDEx study was a double blind, randomized, placebo-controlled trial of up 48 months which sought to investigate whether rivastigmine delayed progression to AD in patients with MCI, and to assess possible benefits among cognitive functions in MCI patients. Subjects were assigned to a rivastigmine group (n=508) or placebo group (n=510). No significant difference in rates were found in either dementia or cognitive benefit, along with more adverse events in the active group.^40^

### Galantamine

In two studies involving 2048 subjects who were allocated to placebo or active groups, galantamine failed to prevent the progression of MCI to dementia. These studies showed no significant changes in ADAS-Cog/MCI or ADCS/MCI scores at month 12 or month 24, irrespective of number of ApoE-4 alleles. Adverse events were more common in the galantamine group.^41^

Current evidence is insufficient to recommend the prescription of cholinesterase inhibitors for MCI or as a measure to delay development of dementia. Several points however should be emphasized: a significant percentage of subjects with MCI, spontaneously revert to normal cognition, and not all cases of MCI progress to dementia; amnestic MCI subjects are more prone to convert to AD than non-amnestic MCI; the instruments available probably lack the sensitivity to measure an effect of these types of drugs.

## Conclusions

Although our review was not systematic, several conclusions can be drawn. Having a healthy lifestyle; physical, cognitive, leisure activities; good social engagement; high consumption of fish, low consumption of dietary fat and moderate consumption of wine; control of vascular risk factors, such as arterial hypertension, diabetes mellitus, dyslipidemias, and being a non-smoker seem to be potential advantages in delaying dementia. These factors are all modifiable in the lives of individuals, and can lead to improved quality of life for all. Other bio and genetic markers may prove useful for determining who could most benefit from these interventions, as well as other pharmacological interventions.

## References

[1] Ferri CP, Prince M, Brayne C (2005). Global prevalence of dementia: a Delphi consensus
study. Lancet.

[2] Nitrini R, Bottino CMC, Albala C (2009). Prevalence of dementia in Latin America: a collaborative study of
population-based cohorts. Int Psychogeriatrics.

[3] Barnes DE, Cauley JA, Lui LY (2007). Women who maintain optimal cognitive function into old
age. J Am Geriatr Soc.

[4] Yaffe K, Fiocco AJ, Vittinghoff E (2009). Predictors of maintaining cognitive function in older adults: The
Health ABC Study. Neurology.

[5] Erten-Lyons D, Woltjer RL, Dodge H (2009). Factors associated with resistance to dementia despite high
Alzheimer disease pathology. Neurology.

[6] Scarmeas N, Levy G, Tang MX, Manly J, Stern Y (2001). Influence of leisure activity on the incidence of Alzheimer's
disease. Neurology.

[7] Wilson RS, Mendes De Leon CF, Barnes LL (2002). Participation in cognitive stimulating activities and risk of
incident Alzheimer's disease. JAMA.

[8] Verghese J, Lipton RB, Katz MJ (2003). Leisure activities and the risk of dementia in the
elderly. N Engl J Med.

[9] Verghese J, LeValley A, Derby C (2006). Leisure activities and the risk of Amnestic Mild Cognitive
Impairment in the elderly. Neurology.

[10] Carlson MC, Helms MJ, Steffens DC, Burke JR, Potter GG, Plassman BL (2008). Midlife activity predicts risk of dementia in older male twin
pairs. Alzheimer's & Dementia.

[11] Bennett DA, Schneider JA, Tang Y, Arnold SE, Wilson RS (2006). The effect of social networks on the relation between Alzheimer's
disease pathology and level of cognitive function in old people: a
longitudinal cohort study. Lancet Neurol.

[12] Podewills LJ, Guallar E, Kuller LH (2005). Physical activity, APOE genotype, and dementia risk: findings
from the Cardiovascular Health Cognition Study. Am J Epidemiol.

[13] Saczynski JS, Pfeifer LA, Masaki K (2006). The effect of social engagement on incident dementia: the
Honolulu-Asia Aging Study. Am J Epidemiol.

[14] Wang HX, Karp A, Winblad B, Fratiglioni L (2002). Late-life engagement in social and leisure activities is
associated with a decreased risk of dementia: a longitudinal study from the
Kungsholmen project. Am J Epidemiol.

[15] Lautenschlager NT, Cox KL, Flicker L (2008). Effect of physical activity on cognitive function in older adults
at risk for Alzheimer's disease. A randomized trial. JAMA.

[16] Uda M, Ishido M, Kami K, Musuhara M (2006). Effects of chronic treadmill running on neurogenesis in the
dentate gyrus of the hippocampus of adult rat. Brain Res.

[17] Ruitenberg A, van Sweiten JC, Witteman JCM (2002). Alcohol consumption and risk of dementia: the Rotterdam
study. Lancet.

[18] Mukamal KJ, Kuller LH, Fitzpatrick AL (2003). Prospective study of alcohol consumption and risk of dementia in
older adults. JAMA.

[18] Espeland MA, Gu L, Masaki KH (2005). Association between reported alcohol intake and cognition:
results from the Women's Health Initiative Memory Study. Am J Epidemiol.

[19] Collins MA, Neafsey EJ, Mukamal KJ (2009). Alcohol in moderation, cardioprotection, and neuroprotection:
epidemiological considerations and mechanistic studies. Alcohol Clin Exp Res.

[20] Mehlig K, Skoog I, Guo X (2008). Alcoholic beverages and incidence of dementia:34-year follow-up
of the prospective population study of women in
Göteborg. Am J Epidemiol.

[21] Marambaud P, Zhao H, Davies P (2005). Resveratrol promotes clearance of Alzheimer's disease amyloid-
β peptides. J Biol Chem.

[22] Vingtdeux V, Dreses-Werringloer U, Zhao H, Davies P, Marambaud P (2008). Therapeutic potential of resveratrol in Alzheimer's
disease. BMC Neuroscience.

[23] Scarmeas N, Stern Y, Mayeux R, Luchsinger JA (2006). Mediterranean diet, Alzheimer disease, and vascular
mediation. Arch Neurol.

[24] Scarmeas N, Stern Y, Tang MX, Mayeux R, Luchsinger JA (2006). Mediterranean diet and risk for Alzheimer's
disease. Ann Neurol.

[25] Scarmeas N, Sterm Y, Mayeux R, Manly JJ, Schupf N, Luchsinger JA (2009). Mediterranean diet and Mild Cognitive Impairment. Arch Neurol.

[26] DeKosky ST, Williamson JD, Fitzpatrick AL (2008). Ginkgo Biloba for prevention of dementia: a randomized controlled
trial. JAMA.

[27] Green RC, McNagny SE, Jayakumar P (2006). Statin use and the risk of Alzheimer's disease: the MIRAGE
Study. Alzheimer's & Dementia.

[28] Cramer C, Haan MN, Galea S (2008). Use of statin and incidence of dementia and cognitive impairment
without dementia in a cohort study. Neurology.

[29] Sparks DL, Kryscio RJ, Sabbagh MN (2008). Reduced risk of incident AD with elective statin use in a
clinical trial cohort. Curr Alzheimer Res.

[30] Haag MDM, Hofman A, Koudstaal PJ, Stricker BHC, Breteler MMB (2009). Statins are associated with a reduced risk of Alzheimer disease
regardless of lipophilicity. The Rotterdam Study. J Neurol Neurosurg Psychiatry.

[31] Hoffman LB, Schmeidler J, Lesser GT (2009). Less Alzheimer disease neuropathology in medicated hypertensive
than non-hypertensive persons. Neurology.

[32] Haag MDM, Hofman A, Koudstaal PJ, Breteler MMB, Stricker BHC (2009). Duration of antihypertensive drug use and risk of dementia: a
prospective cohort study. Neurology.

[33] Hanon O, Forette F (2005). Treatment of hypertension and prevention of
dementia. Alzheimer Dement J Alzheimer Assoc.

[34] Forette F, Seux ML, Staessen JA (1998). Prevention of dementia in randomised double-blind
placebo-controlled Systolic Hypertension in Europe (Syst-Eur)
trial. Lancet.

[35] Tzourio C, Anderson C, Chapman N (2003). Effects of blood pressure lowering with perindopril and
indapamide therapy on dementia and cognitive decline in patients with
cerebrovascular disease. Arch Intern Med.

[36] Petersen RC, Thomas RG, Grundman M (2005). Vitamin E and donepezil for the treatment of mild cognitive
impairment: Alzheimer's Disease Cooperative Study Group. N Engl J Med.

[37] Lu PH, Edland SD, Teng E (2009). Donepezil delays progression to AD in MCI subjects with
depressive symptoms. Neurology.

[38] Doody RS, Ferris SH, Salloway S (2009). Donepezil treatment of patients with MCI - A 48-week randomized,
placebo-controlled trial. Neurology.

[39] Salloway S, Ferris S, Kluger A (2004). Efficacy of donepezil in mild cognitive impairment. A randomized
placebo-controlled trial. Neurology.

[40] Feldman HF, Ferris S, Winblad B (2007). Effect of rivastigmine on delay to diagnosis of Alzheimer's
disease from mild cognitive impairment: the InDDEx study. Lancet Neurol.

[41] Winblad B, Gauthier S, Scinto L (2008). Safety and efficacy of galantamine in subjects with mild
cognitive impairment. Neurology.

